# Nodular cutaneous metastasis of the leg in advanced urothelial bladder carcinoma: a case report and systematic literature review

**DOI:** 10.3389/fonc.2023.1216725

**Published:** 2023-08-22

**Authors:** Paolo Izzo, Luciano Izzo, Silvia Lai, Giuliano D’Onghia, Paola Giancontieri, Raimondo Gabriele, Andrea Polistena, Simone Sibio, Maria Ludovica Costanzo, Claudia De Intinis, Sara Izzo

**Affiliations:** ^1^ “Pietro Valdoni” Department of Surgery, Policlinico “Umberto I”, “Sapienza” University of Rome, Rome, Italy; ^2^ Department of Translational and Precision Medicine, Nephrology Unit, Sapienza University of Rome, Rome, Italy; ^3^ Department of Radiological, Oncological and Anatomo-Pathological Sciences, Policlinico “Umberto I”, “Sapienza” University of Rome, Rome, Italy; ^4^ Multidisciplinary Department of Medical-Surgical and Dental Specialties, Plastic Surgery Unit, Università degli Studi della Campania “Luigi Vanvitelli”, Piazza Luigi Miraglia, Naples, Italy

**Keywords:** bladder, cancer, metastasis, urothelial, cutaneous, cutaneous metastasis, urothelial bladder carcinoma, systematic literature review

## Abstract

Cutaneous metastases from urothelial carcinoma (UC) are very rare and indicate advanced disease with a poor prognosis. A 63-year-old female patient with a history of urothelial bladder carcinoma, treated 2 months prior with radical cystectomy and adjuvant gemcitabine and cisplatin (GC) therapy, presented a skin lesion localized in the lower third of the right leg. Punch biopsy revealed carcinomatous metastasis whose urothelial origin was confirmed by immunohistochemical analysis. 18-FDG PET-CT showed the spread of metastases to the lung and left ischium. Our review focuses on the time between surgery and skin metastasis, localization, and prognosis after metastasis diagnosis. In many cases, skin metastases occur within one year of initial UC surgery and in most cases occur on the abdominal wall. Local wide excision of metastasis should be considered in selected cases; however, chemotherapy remains the main treatment.

## Introduction

Urothelial carcinoma (UC), also known as transitional cell carcinoma (TCC), represents the predominant histological type of all bladder cancers, accounting for approximately 90%. It is the ninth most common cancer in the world (1, 2).

The 5-year survival rate for urothelial carcinoma of the bladder is 77%. While the 5-year survival rate is 96% for “in situ” diagnosed cases, the survival rate falls down to only 4.6% for metastatic cancer (5% of all diagnosed cases) (3).

The most common metastases sites of UC include the lymph node, bone, liver, and lung (4), while UBC metastasis to the skin is a rare occurrence (5).

We present a rare case of urothelial bladder carcinoma with cutaneous metastases localized in the lower third of the right leg, rare because of its nodular clinical form.

The purpose of this study is to identify the mean time within which skin metastasis can occur after surgery for urothelial carcinoma, the most frequent location of skin metastasis, and survival from the diagnosis of skin metastasis. The uncommon occurrence and poor prognosis of cutaneous metastases in UC lead to unclear management and treatment protocols for these patients.

## Methods and case report

**Figure 1:**
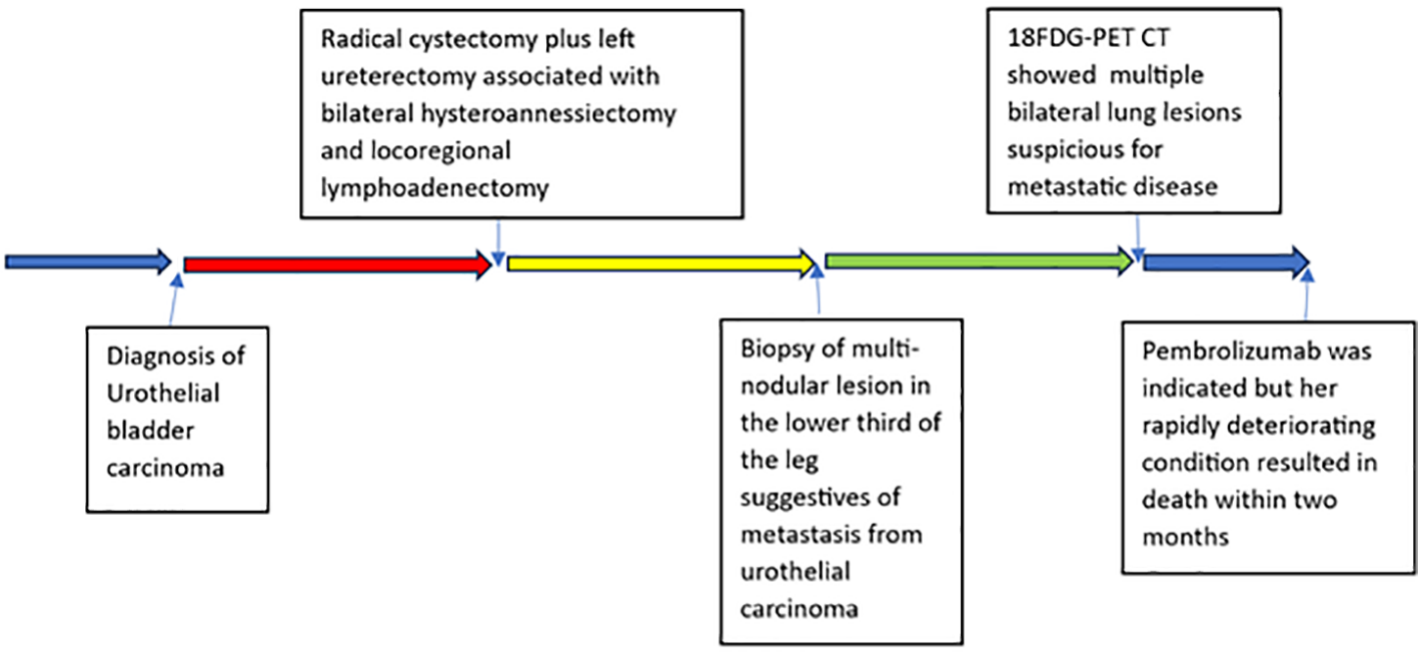
Timeline of the patient’s clinical course with major clinical events.

A 63-year-old woman treated at Policlinico Umberto I – Rome in 2020 is described. Consensus-based clinical case report (CARE) guidelines were applied to present the case report (6). The patient provided written informed consent for the publication. A timeline with relevant data is shown in **Figure 1**.

A systematic review of the online database MEDLINE/PubMed, using the search algorithm [(urothelial OR transitional OR bladder) AND metastasis AND (carcinoma OR cancer) AND (skin OR cutaneous), year > 2000], was carried out. The references in each identified article were reviewed to find additional published case reports.

The identified articles were further processed according to the preferred reporting items for systematic reviews and meta-analyses (PRISMA checklist) (7) in order to identify original case reports of cutaneous metastasis of UC after surgery in the last 22 years.

### Case report

**Figure 2:**
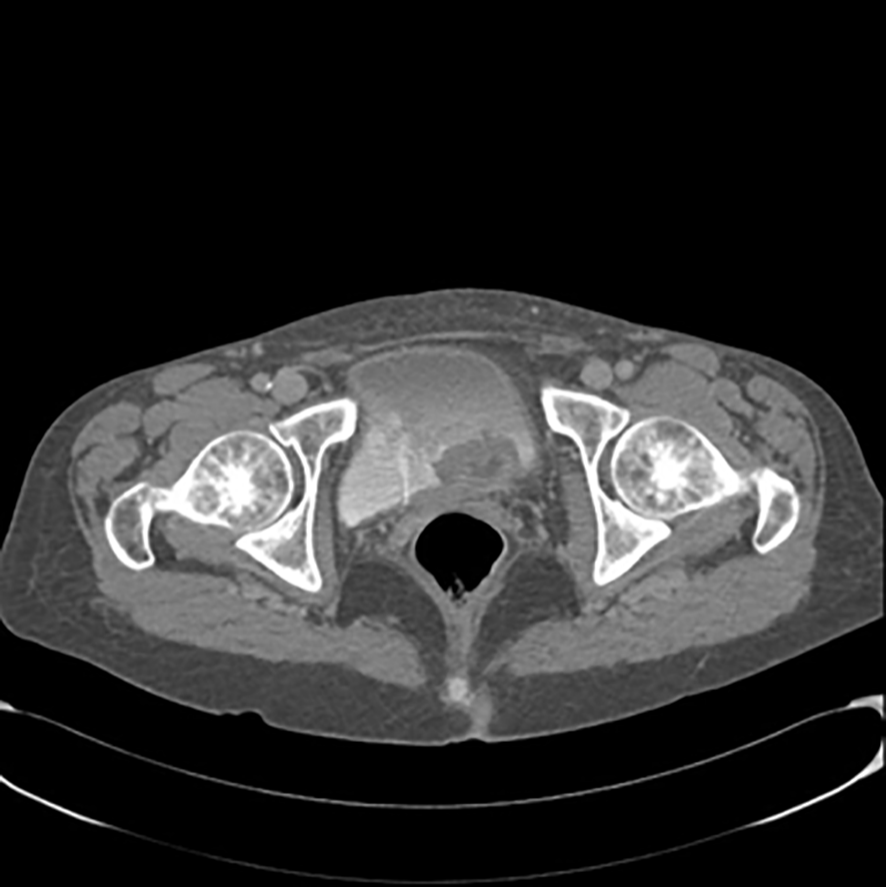
Contrast-enhanced computed tomography scan of the pelvis showing lesions in trigone of bladder extending up to the left ureteral ostium.

A 63-year-old woman presented with a 2–3 month history of hematuria and anemia. Her chronic illness included hypertension and chronic renal failure. Her ECOG performance score was 1. Ultrasonography showed left hydronephrosis. An abdominal-pelvis CT scan (**Figure 2**) and multiparametric magnetic resonance imaging (mpMRI) for bladder cancer showed a VI-RADS score of 5 and tumoral lesions in the trigone of the bladder extending up to the left ureteral ostium, with no lymphadenopathies detected. Clinicians performed transurethral resection of the bladder tumor (TURBT) as the treatment. Pathological diagnosis of the TURBTS showed high grade muscle-invasive urothelial carcinoma. A total body CT was obtained for radiological staging, and this did not show metastatic disease. The patient underwent a radical cystectomy and left ureterectomy associated with bilateral hysteroannessiectomy and locoregional lymphoadenectomy.

Postoperative pathological results revealed urothelial carcinoma pT4a pN1 (1/36), R0. Due to the creatinine value (1.5 mg/dl, eGFR <50 mL/min) and comorbidities, CARBOplatin instead of cisplatin-based chemotherapy was administered. Gemcitabine (1000 mg/m^2^) was administered on day 1 and day 8 and CARBOplatin (AUC5) on day 1 of a 21-day cycle for 4 cycles.

**Figure 3:**
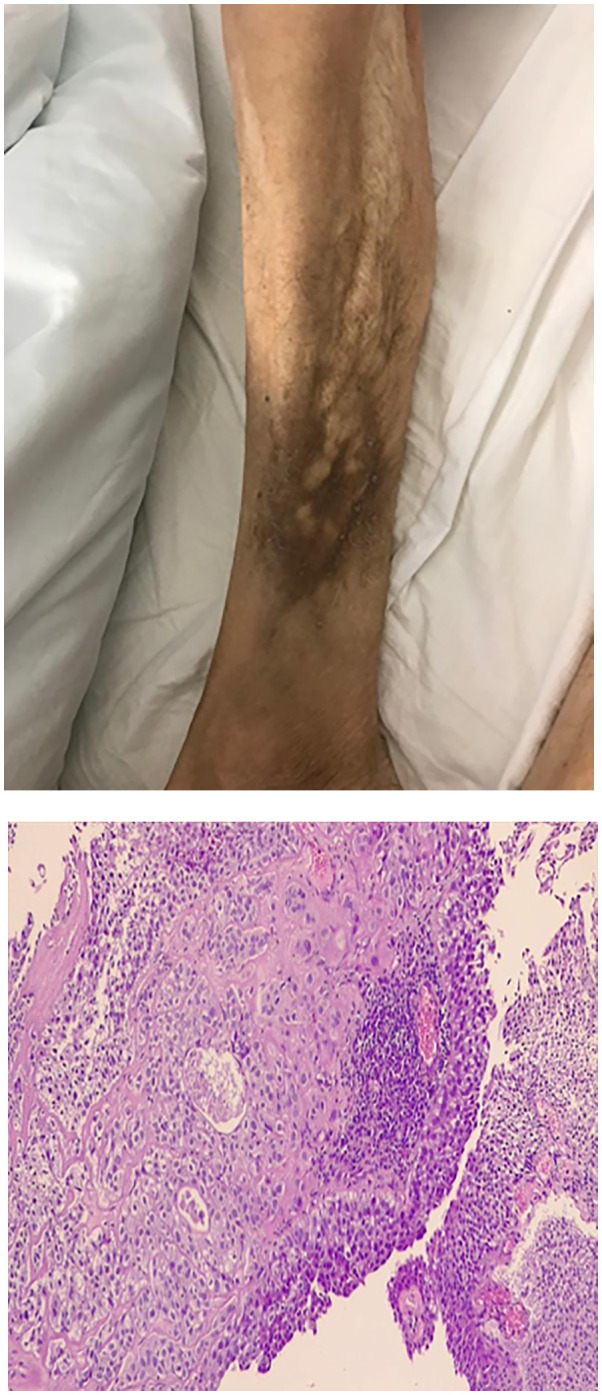
Enlarged lower third of the leg. Clinical appearance of multi-nodular lesion, biopsy showed cutaneous metastasis urothelial carcinoma. Microscopic view of biopsy specimen shows subcutaneous infiltration of urothelial cell carcinoma.

Two months after radical cystectomy, a multi-nodular lesion associated with skin rash in the lower third of the leg appeared (**Figure 3**). The lesion was not painful. A skin biopsy of the leg lesion was performed, showing poorly differentiated carcinomatous proliferation with extensive infiltration from atypical pleomorphic cells with round nuclei and a moderate amount of cytoplasm (**Figure 3**). The following immunohistochemical markers were performed: CK7, CK20, and GATA3. Positivity was suggestive of metastasis from urothelial carcinoma. Furthermore, 18FDG-PET CT showed multiple bilateral lung lesions, suspicious for metastatic disease. Immunotherapy with Pembrolizumab was performed; however, her rapidly deteriorating condition resulted in death within 2 months of diagnosis.

## Review of the literature

A systematic review of the online database MEDLINE/PubMed yielded 49 articles. Twenty-seven articles not exactly matching the aim of the study (patients who did not undergo surgical treatment of a primary tumor; non transitional cell carcinoma histological type; metastatic disease at first diagnosis of UC) were excluded.

The remaining articles consisted of 22 original case reports of cutaneous metastases of urothelial carcinoma in patients who have undergone surgery. The main characteristics of the published cases are summarized in **Table 1**.

**Table 1:** Characteristics of published case reports of cutaneous metastasis from urothelial carcinoma in patients who have undergone surgery for a primary tumor in the last 22 years.

Year	Study	Site	Sex Age	Surgical Treatment +\- Systemic Treatment	Time after surgery; Treatment	Survival time from diagnosis of cutaneous metastases
2022	Cutaneous metastasis of bladder urothelial carcinoma; a rare conditions (8)	Abdomen	Female 80	Cystectomy plus Urethrectomy. Adjuvant Immunotherapy	2 months	–
2021	Urothelial carcinoma of the bladder with cutaneous metastases after robot-assisted radical cystectomy. Case report (9)	Left chest	Male 70	Neoadjuvant chemotherapy and Robot-Assisted laparoscopic radical cystoprostatectomy.	2 months; immunotherapy	Alive after 5 months
2020	A patient with history of bladder cancer presenting with an axillary mass: A rare case of supradiaphragmatic cutaneous bladder cancer metastasis (10)	Axillary metastasis	Male 71	Cystoprostatectomy	5 months	–
2019	Multiple cutaneous metastasis of synchronous urothelial carcinoma of the bladder and the renal pelvis: a case report (11)	Hypochondriac, the back and the cervical region	Male 61	radical cystectomy and a left ureteronephrectomy ➔ adjuvant chemotherapy	12 months	1 month
2017	Metastatic urothelial carcinoma presenting as carcinoma erysipeloides (12)	Bilateral inguinal and suprapubic painful rash	Male 61	Cystoprostatectomy	3 years; chemotherapy	1 month
2016	Transitional bladder cell carcinoma spreading to the skin (13).	Multiple subcutaneous chest lesions	Male 74	cysto-prostatectomy + adjuvant chemotherapy	4 months	–
2015	Metastatic transitional cell carcinoma presenting with skin metastasis (14)	Right hypogastric region	Female 60	Nephroureterectomy	1 year; metastasectomy, chemotherapy	3 months
2015	Cutaneous metastasis of transitional cell carcinoma of the urinary bladder eight years after the primary: a case report (15).	Left lower limb and anterior thigh	Male 81	Cystoprostatectomy	8 years; Radiotherapy	6 months
2015	Cutaneous metastasis of micropapillary urothelial carcinoma (16).	Sovrapubic area	Male 59	Radical nephroureerectomy + chemotherapy	2 months	–
2014	Malignant priapism secondary to isolated penile metastasis from a renal pelvic carcinoma (17).	Penile metastasis	Male 69	Radical resection of the renal pelvic carcinoma	2 months	–
2014	Choroidal and cutaneous metastasis from urothelial carcinoma of the bladder after radical cystectomy: a case report and literature review (18).	Head and lower abdomen	Male 48	Radical cystectomy	17 months	5 months
2014	Cutaneous metastasis of transitional cell carcinoma of the urinary bladder: Cytological aspect (19)	Subscapular, umbilical region and right shoulder	Male 51	transurethral resection of bladder tumor	10 months	–
2012	Cutaneous and subcutaneous metastases from bladder carcinoma (20).	Sovrapubic region	Male 60	Cystoprostatectomy	1 months; Chemotherapy	–
2011	Brain and skin metastasis from urothelial carcinoma of the bladder (21)	Abdominal skin	Male 60	Radical cistectomy	10 years; Chemotherapy	Several days
2011	Cutaneous metastasis of transitional cell bladder carcinoma: a rare presentation and literature review (22).	Lower abdomen	Male 68	Radical cystectomy and bilateral ureterostomy	14 months	2 months
2010	Superficially invasive transitional cell carcinoma of the bladder associated with distant cutaneous metastases (23)	Mid upper back	Male 69	Resected transurethrally	3 months	Several weeks
2006	Skin metastasis of ‘nested type’ of urothelial carcinoma of the urinary bladder (24).	Neck	Male 68	Radical cystoprostatovesiculectomy ➔ chemotherapy	3 years	–
2004	Penile metastasis from primary transitional cell carcinoma of the renal pelvis: first manifestation of systemic spread (25).	Penile metastasis	Male 76	Nephroureterectomy	8 years	–
2004	Twenty-three years of disease-free survival following cutaneous metastasis from a primary bladder transitional cell carcinoma (26).	Skin over the left iliac fossa	Male 51	Cystoprostatourethrectomy	3 months; Wide local excision	Alive 23 years later
2003	Extensive cutaneous metastasis of transitional cell carcinoma of the bladder (27)	Trunk	Male 78	Transurethral resection	6 months	10 months
2003	Transitional cell carcinoma metastasis to arm skin from the renal pelvis (28).	Bilateral arm nodules	Female 68	Right nephroureterectomy	18 months	1 month
2000	Metastasis to the male breast from carcinoma of the urinary bladder (29).	Breast	Male 69	Transurethral resection	2 months	–

## Discussion

Skin metastases from solid primary tumors are rare. The most frequent primary tumors metastasizing to the skin are subject to the gender of the patient and are most common for breast (69%) followed by colon (9%), lung (4%), and ovary (4%) tumors in women and lung (24%) followed by colon (19%) and head and neck malignancy (12%) in men (30).

Mueller et al. (31), in a 2004 review, identify 2,369 cases of dermatologic metastases arising from 81,618 primary solid malignancies. Cutaneous metastases from primary urologic malignancies of the bladder, prostate, or kidney were noted in 116 (1.1%) of 10,417 cases. Of these, 63 cases (3.4%) of metastatic renal cell carcinoma from 1,877 cases were noted, and 38 cases (0.84%) of cutaneous metastatic transitional cell carcinoma (TCC) from 4,516 cases have been reported.

The most common location of metastasis of transitional cell carcinoma disease are known to be the bones, lungs, and the liver (32). However, skin metastasis of transitional cell carcinoma of the bladder is very rare, representing only 0.84% of all cutaneous metastases (19).

There are four recognized mechanisms that can explain a tumor spreading to the skin: hematogenous spread, lymphatics spread, direct invasion, and iatrogenic implantation during surgical treatment (33).

The clinical appearance of skin metastases from UC should mimic many other common dermatologic illnesses (34); therefore, correct diagnosis requires a lesion biopsy with histological examination to establish the urothelial beginning of these skin lesions (28). Metastatic lesions do not often preserve histologic similarities to the primary lesion; in these cases, immunohistochemical examination is mandatory for correct diagnosis and management. Nevertheless, in one case report, a fine needle aspiration biopsy was carried out in order to establish definitive diagnosis (35).

In our patient, skin metastasis affected the lower limb 2 months after the initial diagnosis of UC. Eleven cases found in the literature presented metastases localized on the abdominal wall, while two involved the penis. Seven cases involved chest skin, of which two presented abdominal cutaneous lesions at the same time. Only one case reported axillary skin metastasis. One case showed cutaneous metastases to the lower limb, one to the upper arm and one involved the skin of the neck region. Therefore, in the majority of cases, cutaneous metastases develop in the locoregional skin, spreading through the lymphatic channels (36, 37) or via iatrogenic implantation in surgery (14, 38)

Our patient was a 63-year-old woman; the cases reported in the literature showed a median age of 69 years in female patients. Most cases (19/22) were men with a median age of 65.

The systematic review showed that in 11 cases, the metastases occurred within 6 months of surgery, of which five emerged during adjuvant chemotherapy. Four cases presented skin metastases between 6 and 12 months after surgery. In seven cases, skin metastases were found more than 1 year after surgery, of which two cases were administered adjuvant chemotherapy.

Our patient expired within two months of the diagnosis of UC cutaneous metastasis. Only 12 cases (12/22) reported the evolution from the diagnosis of cutaneous spread from UC. Eleven cases showed a poor prognosis, with a median survival of less than 12 months. One case described a patient alive 23 years after diagnosis of a skin lesion treated with wide surgical excision.

The strength of this article lies in the fact that it is very well documented, describing the patient’s clinical history point-by-point and allowing the reader to have a full understanding of the text, thus hoping that they will get the maximum benefit from this manuscript. However, one must remember the very nature of the article which, as a case report, has obvious limitations, despite a review of the literature.

## Conclusion

Cutaneous dissemination from UC is an extremely rare form of presentation and is a sign of systemic involvement linked to a poor prognosis. Punch biopsy of the lesion should be performed for pathological examination in order to prevent misdiagnosis. Poor outcomes could be attributed to the aggressive nature of the disease, limited treatment options, and rarity of the disease process. Chemotherapy remains the main treatment of choice and metastasectomy should be considered in single-site metastasis.

## Data availability statement

The raw data supporting the conclusions of this article will be made available by the authors, without undue reservation.

## Ethics statement

Written informed consent was obtained from the participant/patient(s) for the publication of this case report.

## Author contributions

Creators of the work: PI, SI, and CDI; Research: GD’O, PG, and LI; For the English translation: SL and MC; Reviewers: AP, RG, and SS. All authors contributed to the article and approved the submitted version.

## References

[1] Ferlay J Ervik M Lam F Colombet M Mery L Piñeros M. Global Cancer Observatory: Cancer Today (2018). Lyon, France: International Agency for Research on Cancer. Available at: https://gco.iarc.fr/ (Accessed 10 January 2020).

[2] Miyazaki J Nishiyama H. Epidemiology of urothelial carcinoma. Int J Urol (2017) 24(10):730–4. doi: 10.1111/iju.13376 28543959

[3] Saginala K Barsouk A Aluru JS Rawla P Padala SA Barsouk A. Epidemiology of bladder cancer. Med Sci (Basel). (2020) 8(1):15. doi: 10.3390/medsci8010015 32183076 PMC7151633

[4] Babaian RJ Johnson DE Llamas L Ayala AG. Metastases from transitional cell carcinoma of urinary bladder. Urology (1980) 16:142–4. doi: 10.1016/0090-4295(80)90067-9 7404907

[5] Teyateeti P Ungtrakul T. Retrospective review of cutaneous metastasis among 11,418 patients with solid Malignancy: A tertiary cancer center experience. Med (Baltimore). (2021) 100(29):e26737. doi: 10.1097/MD.0000000000026737 PMC8294925 34398051

[6] Gagnier JJ Kienle G Altman DG Moher D Sox H Riley D. The CARE guidelines: consensus-based clinical case reporting guideline development. Glob Adv Health Med (2013) 2:38–43. doi: 10.7453/gahmj.2013.008 PMC3833570 24416692

[7] Moher D Liberati A Tetzlaff J Altman DG. Preferred reporting items for systematic reviews and meta-analyses: the PRISMA statement. BMJ (2009) 339:b2535. doi: 10.7326/0003-4819-151-4-200908180-00135 19622551 PMC2714657

[8] Öztürk H Yurtsever S Özer A Arslan Ç Pehlivan FS Tekeli A. Cutaneous metastasis of bladder urothelial carcinoma; A rare conditions. Urol Case Rep (2021) 41:101955. doi: 10.1016/j.eucr.2021.101955 35028295 PMC8739474

[9] Yajima S Nakanishi Y Wantanabe R Matsumoto S Tanabe K Masuda H. Urothelial carcinoma of the bladder with cutaneous metastases after robot-assisted radical cystectomy. Case Rep Urol Case Rep (2021) 38:101709. doi: 10.1016/j.eucr.2021.101709 34040988 PMC8144335

[10] Bhakta PS Harris EA Connolly MM. A patient with history of bladder cancer presenting with an axillary mass: A rare case of supradiaphragmatic cutaneous bladder cancer metastasis. Urol Case Rep (2020) 31:101157. doi: 10.1016/j.eucr.2020.101157 32322508 PMC7163323

[11] Ghalleb M Ayadi MA Slim S Zemni I Doghri R Ben Hassouna J. Multiple cutaneous metastasis of synchronous urothelial carcinoma of the bladder and the renal pelvis: a case report. J Med Case Rep (2019) 13(1):34. doi: 10.1186/s13256-019-1997-8 30760315 PMC6375173

[12] Grace SA Livingood MR Boyd AS. Metastatic urothelial carcinoma presenting as carcinoma erysipeloides. J Cutan Pathol (2017) 44(6):513–5. doi: 10.1111/cup.12953 28425107

[13] Kerkeni W Ayari Y Charfi L Bouzouita A Ayed H Cherif M. Transitional bladder cell carcinoma spreading to the skin. Urol Case Rep (2017) 11:17–8. doi: 10.1016/j.eucr.2016.11.028 PMC5220250 28083478

[14] Açıkgöz O Ölçücüoğlu E Kasap Y Yığman M Güneş ZE Gazel E. Metastatic transitional cell carcinoma presenting with skin metastasis. Int J Crit Illn Inj Sci (2015) 5(1):53–5. doi: 10.4103/2229-5151.152346 PMC4366830 25810966

[15] Lees AN. Cutaneous metastasis of transitional cell carcinoma of the urinary bladder eight years after the primary: a case report. J Med Case Rep (2015) 9:102. doi: 10.1186/s13256-015-0585-9 25943325 PMC4427993

[16] Truong H Parsons TM Trabulsi EJ. Cutaneous metastasis of micropapillary urothelial carcinoma. Urology (2015) 85(2):e7–8. doi: 10.1016/j.urology.2014.10.015 25559725

[17] Liu S Zeng F Qi L Jiang S Tan P Zu X. Malignant priapism secondary to isolated penile metastasis from a renal pelvic carcinoma. Can Urol Assoc J (2014) 8(7-8):E558–60. doi: 10.5489/cuaj.1695 PMC4137026 25210564

[18] Mitsui Y Arichi N Inoue K Hiraki M Nakamura S Hiraoka T. Choroidal and cutaneous metastasis from urothelial carcinoma of the bladder after radical cystectomy: a case report and literature review. Case Rep Urol (2014) 2014:491541. doi: 10.1155/2014/491541 25431734 PMC4241253

[19] Narayana MA Patnayak R Rukmangadha N Chowhan AK Kottu R Phaneendra BV. Cutaneous metastasis of transitional cell carcinoma of the urinary bladder: Cytological aspect. J Cytol (2014) 31(1):50–2. doi: 10.4103/0970-9371.130707 PMC4150344 25190986

[20] Dirican A Küçükzeybek Y Somalι I Erten C Demir L Can A. Cutaneous and subcutaneous metastases from bladder carcinoma. Contemp Oncol (Pozn). (2012) 16(5):451–2. doi: 10.5114/wo.2012.31779 PMC3687452 23788928

[21] Chung JH Lee JY Pyo JY Oh YH Lee SW Moon HS. Brain and skin metastasis from urothelial carcinoma of the bladder. Korean J Urol (2013) 54(1):66–8. doi: 10.4111/kju.2013.54.1.66 PMC3556557 23362451

[22] Salemis NS Gakis C Zografidis A Gourgiotis S. Cutaneous metastasis of transitional cell bladder carcinoma: a rare presentation and literature review. J Cancer Res Ther (2011) 7(2):217–9. doi: 10.4103/0973-1482.82940 21768720

[23] Swick BL Gordon JR. Superficially invasive transitional cell carcinoma of the bladder associated with distant cutaneous metastases. J Cutan Pathol (2010) 37(12):1245–50. doi: 10.1111/j.1600-0560.2009.01471.x 19919656

[24] Zwenzner EM Kaatz M Ziemer M. Skin metastasis of ‘nested type’ of urothelial carcinoma of the urinary bladder. J Cutan Pathol (2006) 33(11):754–5. doi: 10.1111/j.1600-0560.2006.00503.x 17083696

[25] Pomara G Pastina I Simone M Casale P Marchetti G Francesca F. Penile metastasis from primary transitional cell carcinoma of the renal pelvis: first manifestation of systemic spread. BMC Cancer (2004) 4:90. doi: 10.1186/1471-2407-4-90 15575962 PMC539302

[26] Gowardhan B Mathers ME Feggetter JG. Twenty-three years of disease-free survival following cutaneous metastasis from a primary bladder transitional cell carcinoma. Int J Urol (2004) 11(11):1031–2. doi: 10.1111/j.1442-2042.2004.00939.x 15509212

[27] Akman Y Cam K Kavak A Alper M. Extensive cutaneous metastasis of transitional cell carcinoma of the bladder. Int J Urol (2003) 10(2):103–4. doi: 10.1046/j.1442-2042.2003.00571.x 12588608

[28] Lin CY Lee CT Huang JS Chang LC. Transitional cell carcinoma metastasis to arm skin from the renal pelvis. Chang Gung Med J (2003) 26(7):525–9. 14515977

[29] Cappabianca S Grassi R D’Alessandro P Del Vecchio A Maioli A Donofrio V. Metastasis to the male breast from carcinoma of the urinary bladder. Br J Radiol (2000) 73(876):1326–8. doi: 10.1259/bjr.73.876.11205680 11205680

[30] Brownstein MH Helwig EB. Metastatic tumors of the skin. Cancer (1972) 29:1298–307. doi: 10.1002/1097-0142(197205)29:5<1298::AID-CNCR2820290526>3.0.CO;2-6 4336632

[31] Mueller TJ Wu H Greenberg RE Hudes G Topham N Lessin SR. Cutaneous metastases from genitourinary Malignancies. Urology (2004) 63(6):1021–6. doi: 10.1016/j.urology.2004.01.014 15183939

[32] Elston DM Tuthill RJ Pierson J Marden JD Bergfeld WF. Carcinoma erysipelatoides resulting from genitourinary cancer. J Am Acad Dermatol (1996) 35:993–5. doi: 10.1016/S0190-9622(96)90131-0 8959966

[33] Scott LS Head MA Mack WS. Cutaneous metastases from tumours of the bladder, urethra, and penis. Br J Urol (1954) 26:387–400. doi: 10.1111/j.1464-410X.1954.tb04922.x 13219333

[34] Persechino F Franceschini C Cota C Frascione P Ardigò M. A new single red nodule on the abdomen of a wOman with history of endometrial carcinoma: Noninvasive evaluation and histologic correlation. JAAD Case Rep (2018) 4(9):941–3. doi: 10.1016/j.jdcr.2018.07.004 PMC6191948 30345339

[35] Kumar PV Salimi B Musallaye A Tadayyon A. Subcutaneous metastasis from transitional cell carcinoma of the bladder diagnosed by fine needle aspiration biopsy. A Case Rep Acta Cytol (2000) 44:657–60. doi: 10.1159/000328543 10934962

[36] Saito S. Solitary cutaneous metastasis of superficial bladder cancer. Urol Int (1998) 61:126. doi: 10.1159/000030304 9873256

[37] Hollander A Grots IA. Oculocutaneous metastases from carcinoma of the urinary bladder. Case Rep Rev lit Arch Dermatol (1968) 97:678. doi: 10.1001/archderm.1968.01610120068010 4297449

[38] Miyamoto T Ikehara A Araki M Akaeda T Mihara M. Cutaneous metastatic carcinoma of the penis: suspected metastasis implantation from a bladder tumor. J Urol (2000) 163(5):1519. doi: 10.1097/00005392-200005000-00030 10751873

