# Erratum: Nodular cutaneous metastasis of the leg in advanced urothelial bladder carcinoma: a case report and systematic literature review

**DOI:** 10.3389/fonc.2023.1286604

**Published:** 2023-09-27

**Authors:** Frontiers Production Office

**Affiliations:** Frontiers Media SA, Lausanne, Switzerland

**Keywords:** bladder, cancer, metastasis, urothelial, cutaneous, cutaneous metastasis, urothelial bladder carcinoma, systematic literature review

Due to a production error, there was a mistake in the figures as published. **Figure 1** was erroneously excluded, and what is now **Figure 3** was included as two separate figures. The corrected **Figures 1**–**3**, and their captions, appear below.

**Figure 1:**
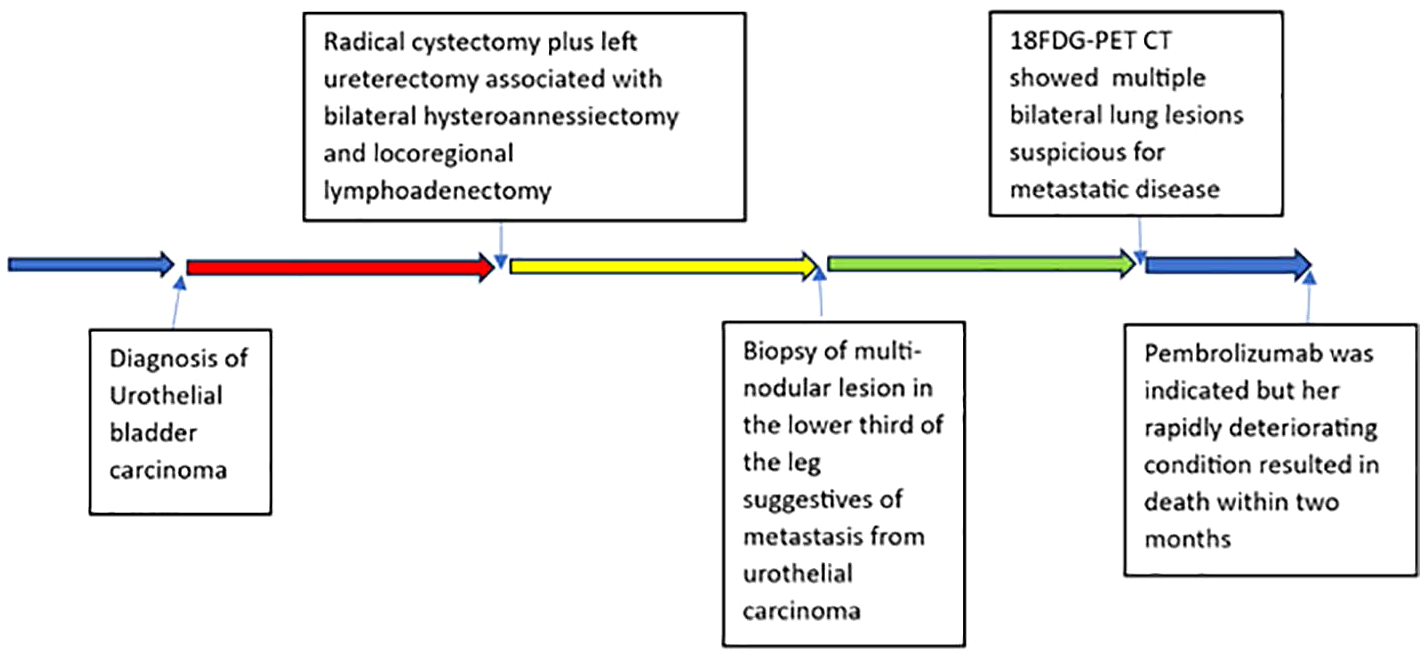
Timeline of the patient’s clinical course with major clinical events.

**Figure 2:**
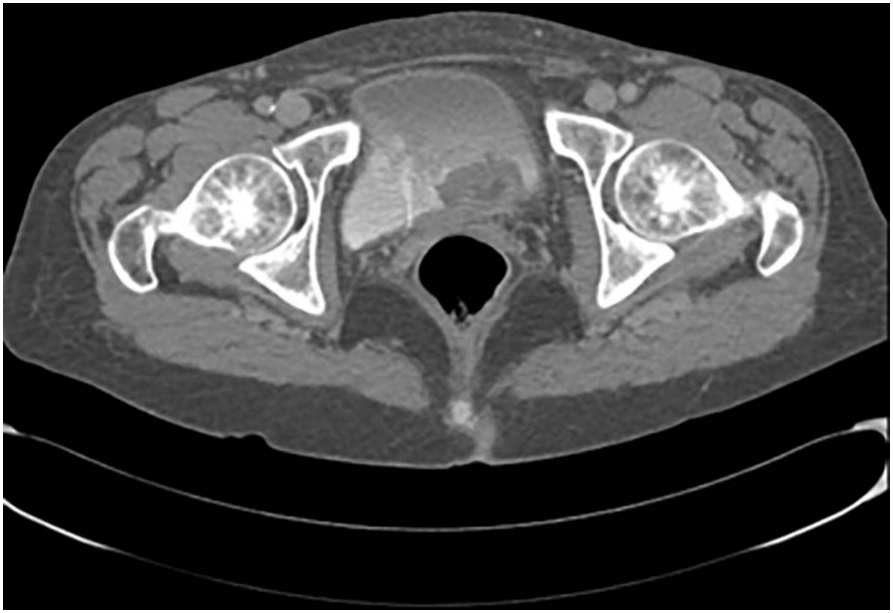
Contrast-enhanced computed tomography scan of the pelvis showing lesions in trigone of bladder extending up to the left ureteral ostium.

**Figure 3:**
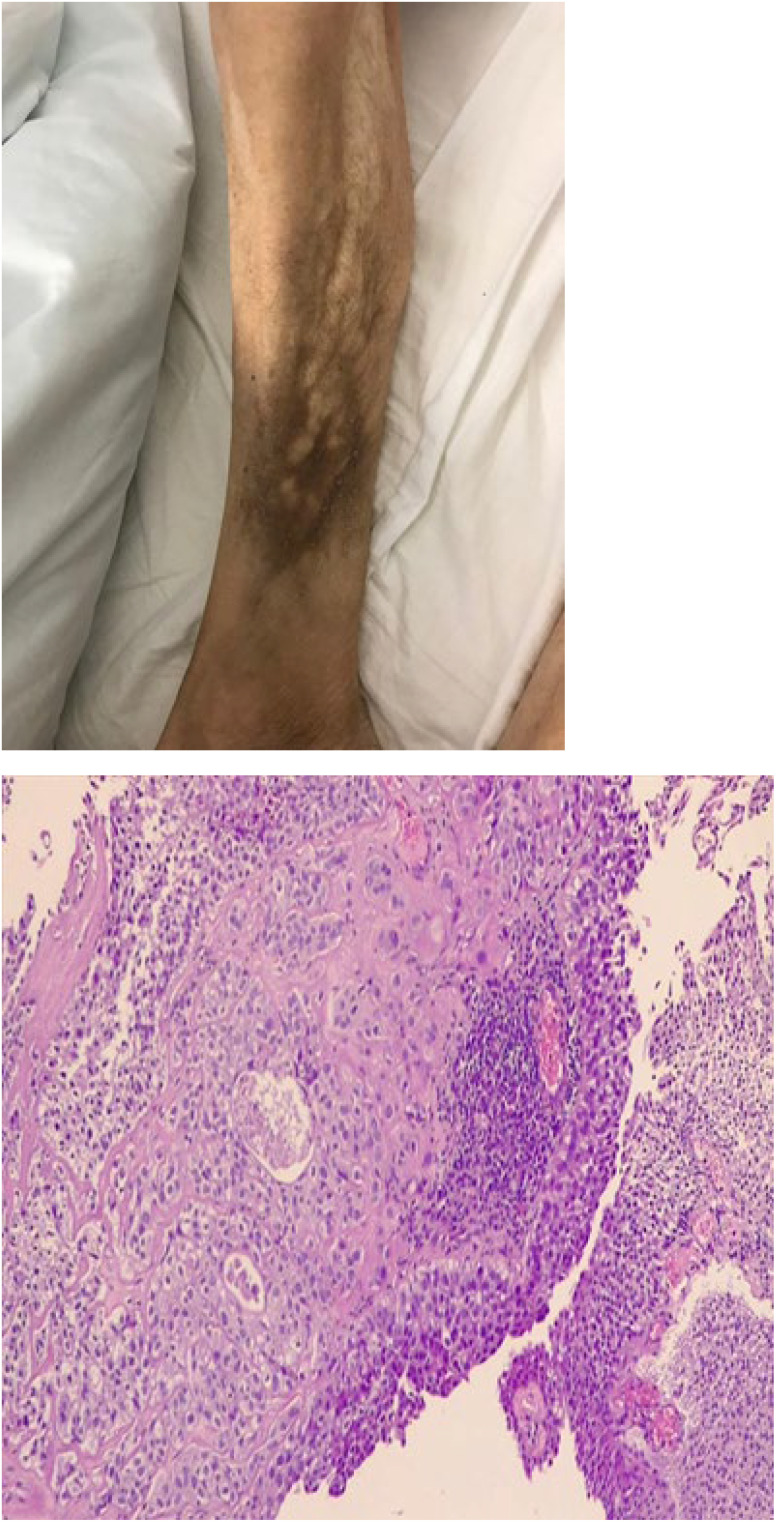
Enlarged lower third of the leg. Clinical appearance of multi-nodular lesion, biopsy showed cutaneous metastasis urothelial carcinoma. Microscopic view of biopsy specimen shows subcutaneous infiltration of urothelial cell carcinoma.

The publisher apologizes for this mistake. The original version of this article has been updated.

